# Supplementary material for: Multiplexed in situ hybridization reveals distinct lineage identities for major and minor vein initiation during maize leaf development
Source: Proc Natl Acad Sci U S A. 2024 Jul 3;121(28):e2402514121. doi: 10.1073/pnas.2402514121 (PMC11252972; doi:10.1073/pnas.2402514121)
Supplement: Supplementary file 1 — Appendix 01 (PDF) [file pnas.2402514121.sapp.pdf]

PNAS

Supporting Information for

Multiplexed *in situ* hybridization reveals distinct lineage identities for major and minor vein initiation during maize leaf development

Chiara Perico, Maricris Zaidem, Olga Sedelnikova, Samik Bhattacharya, Christian Korfhage & Jane A. Langdale

Corresponding author: Jane A. Langdale
Email: jane.langdale@biology.ox.ac.uk

This PDF file includes:

Materials and Methods
Figures S1 to S7
Tables S1 to S3
Legends for Datasets S1 to S4
SI References

Other supporting materials for this manuscript include the following:

Datasets S1 to S4

Materials and Methods

Molecular Cartography probe design
Probes were designed using Resolve BioSciences' proprietary design algorithm and gene annotations from the *Zea Mays* RefGen V4. The final set of probes was selected as outlined in (1).

Plant growth and tissue preparation for Molecular Cartography
Samples were prepared, hybridized and imaged as in Vlad et al. (2). In total six sections were analysed - E1B2, E1D2, S1B1, S1B2, S1A1 and S1A2. Two independent experiments were carried out – the first with sections E1B2 and E1D2 and the second with samples S1A1, S1A2, S1B1 and S1B2.

Data analysis
All data analyses and plots were carried out with R Studio and the Molecular Cartography ImageJ plugin Polylux proprietary of Resolve Biosciences. Unprocessed single-cell data was extracted from the spatial images using the ImageJ plugin Polylux from Resolve BioSciences. Correlation plots were generated using *corrplot* (3) to evaluate the linear relationships between genes across experimental repeats (note that each experimental repeat was also a biological repeat as each section was taken from a different shoot apex). The R package Seurat (4) was used to carry out all clustering data analyses and plotting. The Seurat clustering workflow was adapted with minor adjustments to fit the data frame from molecular cartography. Briefly, for each stage, a Seurat object was generated from unprocessed data with parameters: min.cells = 20 (P2) or 30 (M-P1, P3, P4, M to P5); min.features=3. Parameters were adjusted to account for the difference in the number of cells per primordia stage. Data was normalised using the "LogNormalize" method and a scale factor of 1. Scaling using ScaleData, to prevent overrepresentation of frequent signals in dimensional reductionwas then done using default parameters. A principal component analysis (PCA) was performed using standard parameters. After determining dimensionality of the dataset via the Seurat ElbowPlot function, 10 components were taken into consideration to run the Uniform Manifold Approximation and Projection (UMAP) dimensionality reduction to cluster the cells Clustering was based on neighbours, found using the "FindNeighbours" command (dims = 1:10, dims = 1:13 for M to P5), followed by a default "FindClusters". FindClusters allowed cluster generation by constructing a Shared Nearest Neighbour (SNN) graph and applying the Louvain algorithm. Cluster resolution was set at either 0.3 (P4, M to P5) or 0.4 (M-P1, P2, P3). Dimension plots were generated using the "DimPlot" command and "umap" as the reduction method. Cluster location was visually checked using the Polylux plugin, and identity was assigned based on the cluster localisation within the sections.

Tissue fixation and preparation for traditional in situ hybridization
Traditional *in situ* hybridization was carried out using wax-embedded wild-type B73 maize shoot apices as previously described (5) with a 268 bp digoxygenin (DIG)-RNA labelled probe complementary to the 3'UTR sequence of the *LAX2* gene (starting at 126 bp downstream of the stop codon).

Vein width quantification
Wild-type B73 maize seeds (n = 5) were germinated and grown as for Molecular Cartography, using a 3:1 mixture of John Innes #3 and vermiculite. Following full blade expansion of leaves three and four, 5 mm segments spanning the whole leaf width were harvested from the widest point of the blade. Samples were then fixed in 3:1 EtOH/acetic acid for 30 min at room temperature, followed by a 30 min wash in 70% EtOH. After an overnight incubation in 70% EtOH at 4°C, leaf segments were embedded in blocks of 6% agar and sectioned using a vibratome. Leaf sections were imaged using a stereomicroscope and the width of lateral and intermediate veins outside of the midrib region were measured using ImageJ. Lateral veins were labelled with increasing numbers across the medio-lateral axis (L1 indicating the lateral vein closest to the midrib). Average intermediate veins number and width were measured between successive lateral veins, and between the last lateral vein and the leaf margin. All vein width quantifications were carried out on one half of the leaf segments.

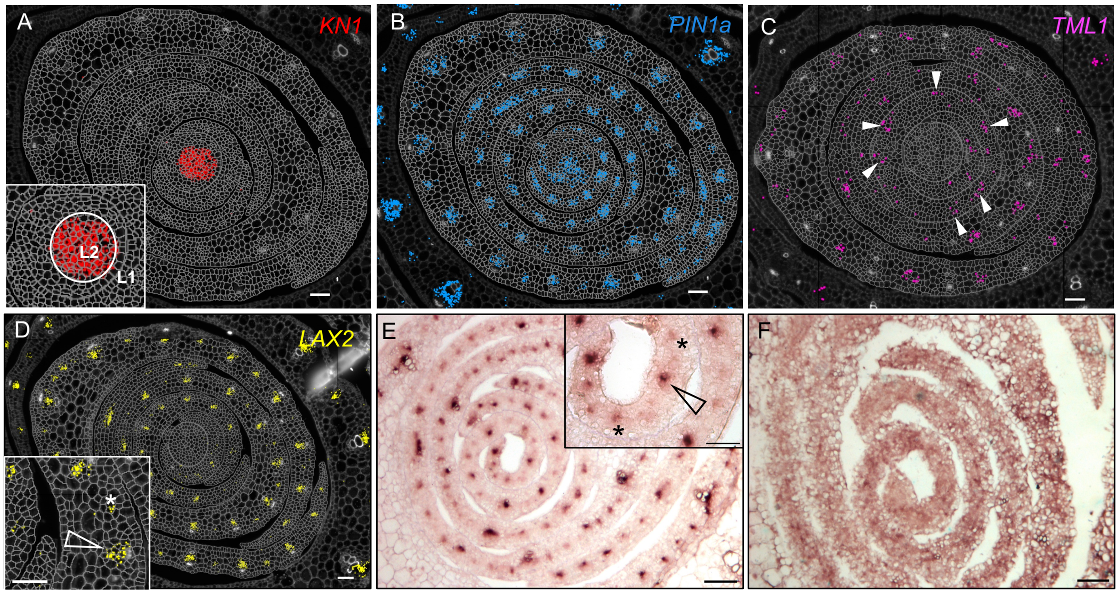

Fig. S1. Validation of Molecular Cartography methodology for localization of transcripts in maize shoot apices. **A)** *KN1* transcripts localize in the shoot apical meristem, specifically the L2 layer as previously shown by in situ hybridization (6). **B)** *PIN1a* transcripts mark all developing veins at all stages of leaf development. **C)** *TML1* transcripts accumulation in developing intermediate veins from stage P3 (white arrowheads). **D, E)** Molecular Cartography (D) and *in situ* hybridization (E) of *LAX2*. In both cases, transcripts are detected in developing vascular tissue with higher levels detected in major veins (inset, arrowhead) compared to minor veins (inset, asterisks). **F)** *In situ* hybridization control with sense *LAX2* probe. Scale bars = 50 μm.

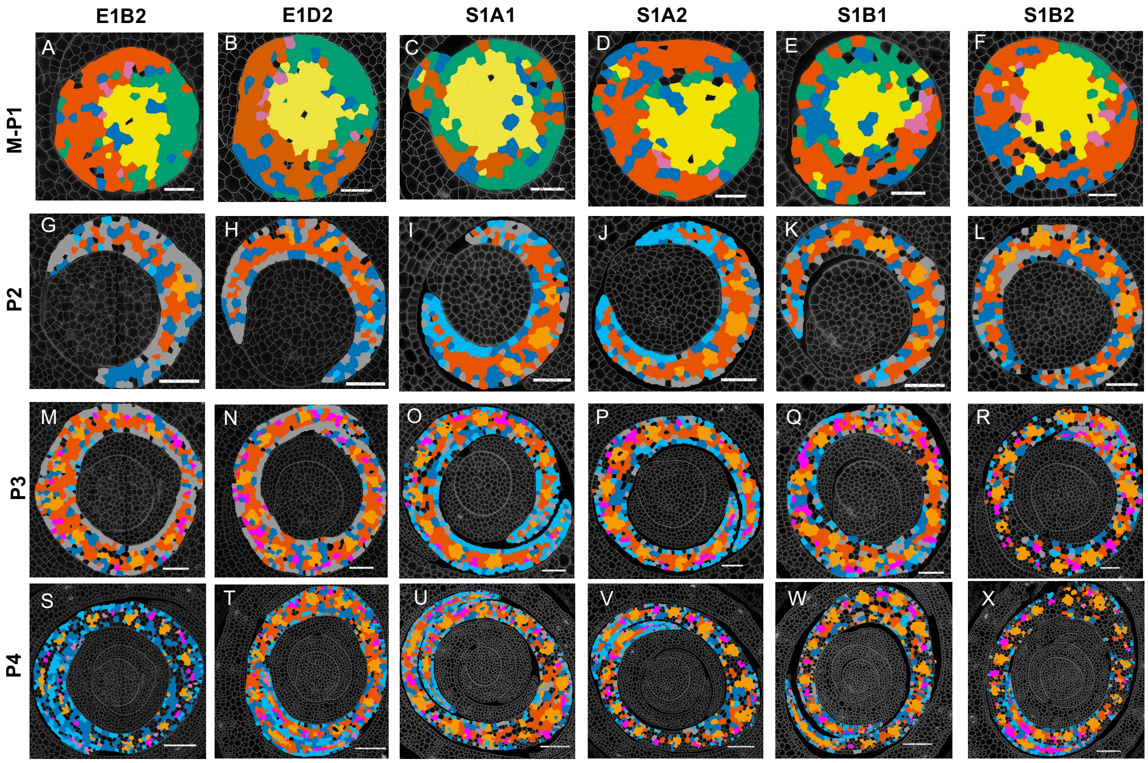

Fig. S2. Visual representation of clusters at all stages of leaf development. Sample numbers are depicted across the top and plastochron stages at the left. Cluster colours are as depicted in the main figures. Scale bars = 30 µm (A-F), 50 µm (G-R), 100 µm (S-X).

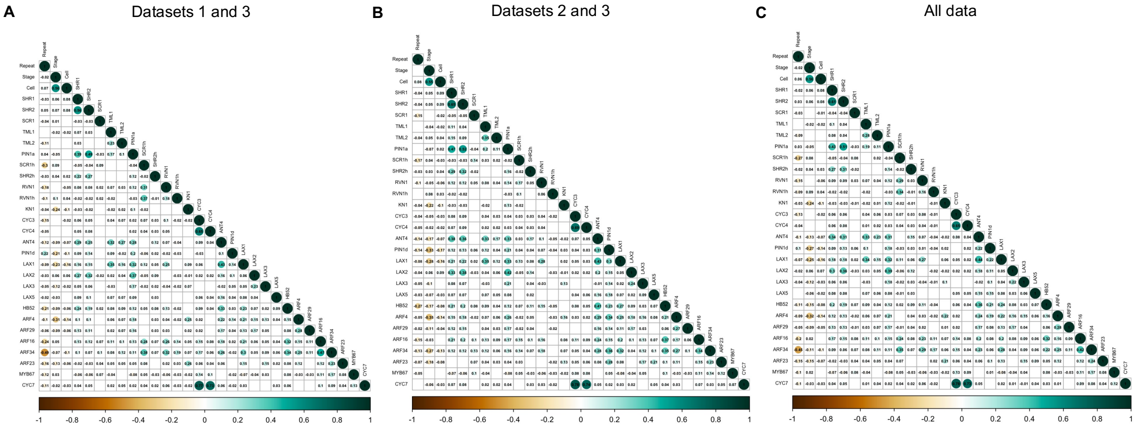

Fig. S3. Correlation analysis of the Molecular Cartography datasets. A) Correlation plot for comparison of different experimental datasets with the same tissue preparation method (dataset 1: E1B2, E1D2; dataset 3: S1B1, S1B2). **B)** Correlation plot for comparison of datasets with manual (dataset 3) and automatic (dataset 2: S1A1, S1A2) tissue preparation method within the same experiment. **C)** All datasets together. Brown colour indicates negative correlations between rows and columns, blue indicates a positive correlation. The number in each cell represents the correlation coefficient (confidence level = 0.95).

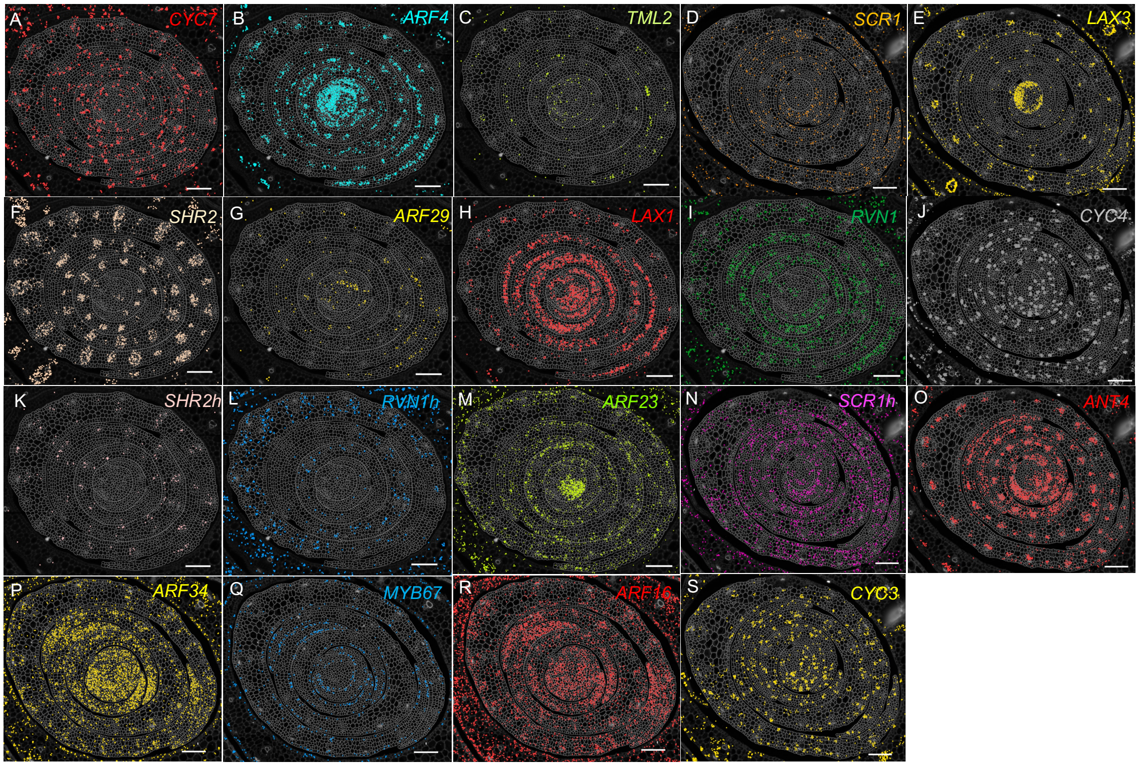

Fig. S4. Representative images showing the localization of transcripts for all genes in the Molecular Cartography panel. Images are chosen across all sections. Representative transcript localization for *PIN1a*, *KN1*, *LAX2*, *SHR1*, *TML1* and *HB52* are provided in Fig. S1, Fig. 6 and Fig. 7. Scale bars = 100 µm.

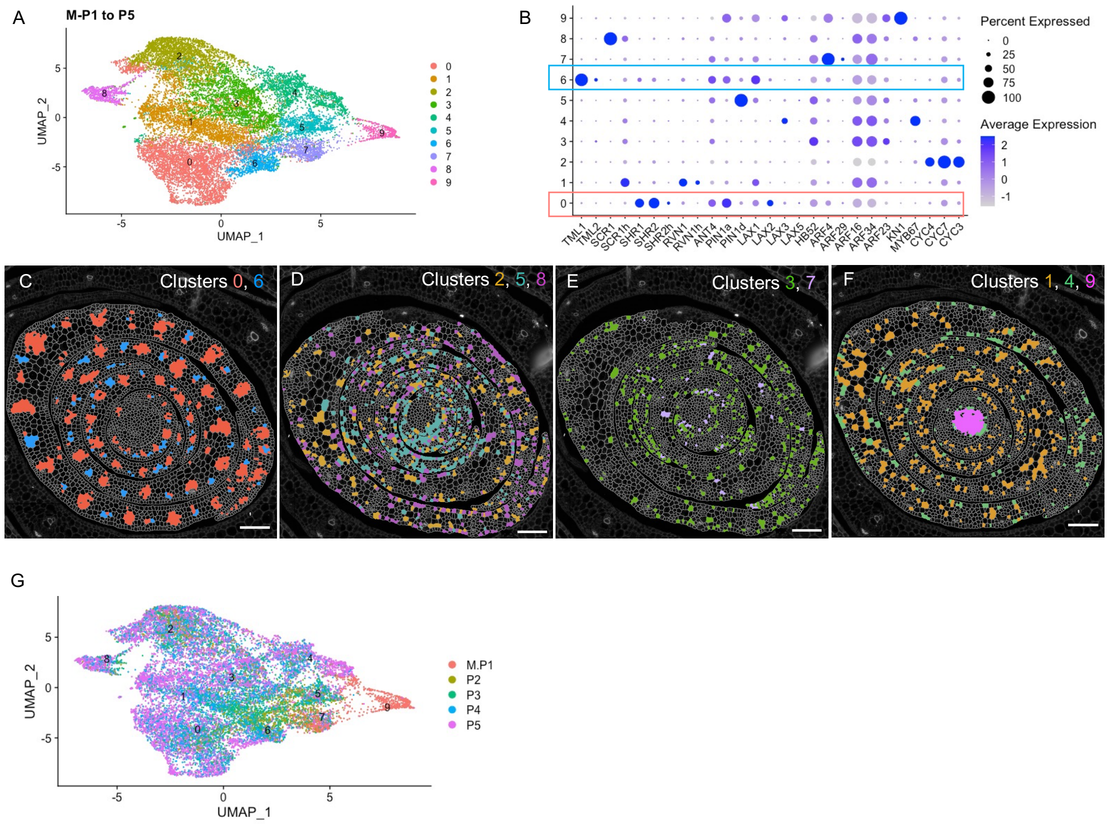

Fig. S5. Data clustering from stage M-P1 to P5 indicates separate identities for major and minor veins. A) Cluster analysis and UMAP plot of data collectively analysed for stage M-P1 to P5. **B)** Dotplot summarising transcript levels for each gene in each cluster at M-P1 to P5. Average expression refers to the average transcription level for each gene in a cluster (cell type), calculated from the scaled data. Percent expressed refers to the percent of cells in a cluster in which transcripts (as indicated on the x-axis) were detected. Blue and red boxes highlight the expression profiles of clusters 6 and 0. **C-F)** Representative images of cluster localisation from M to P5, colour-coded as in panel A. **G)** The UMAP plot in A) is colour-coded to show the contribution of different developmental stages across all clusters. Scale bars = 100 µm.

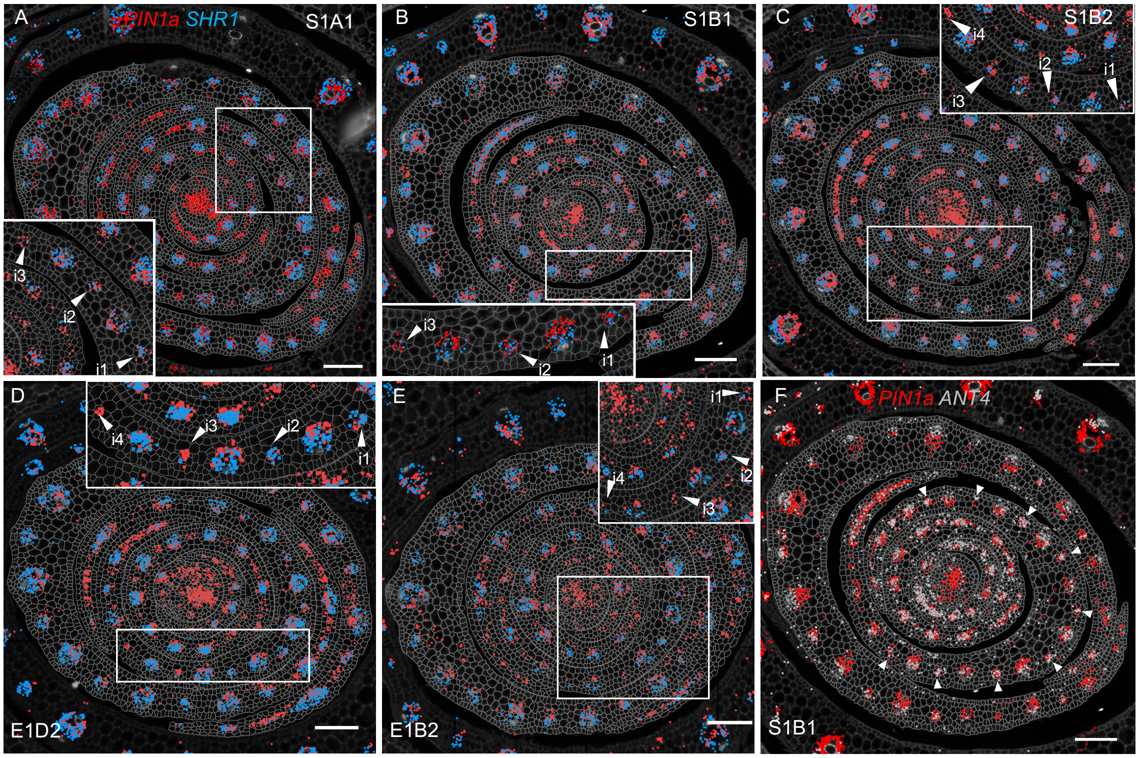

Fig. S6. *PIN1a* expression precedes that of *SHR1* across the medio-lateral axis*. A-E)* Transcript accumulation for *PIN1a* and *SHR1* is detected in both the sheath and blade and in both major and minor veins. Intermediate veins closer to the midrib accumulate *SHR1* before those closer to the leaf margin. In insets, intermediate veins are labelled with progressive numbers indicating the degree of proximity to the midrib. **F)** Unlike *SHR1*, *ANT4* is detected in all intermediate veins across the medio-lateral axis (white arrowheads). Scale bars = 100 µm.

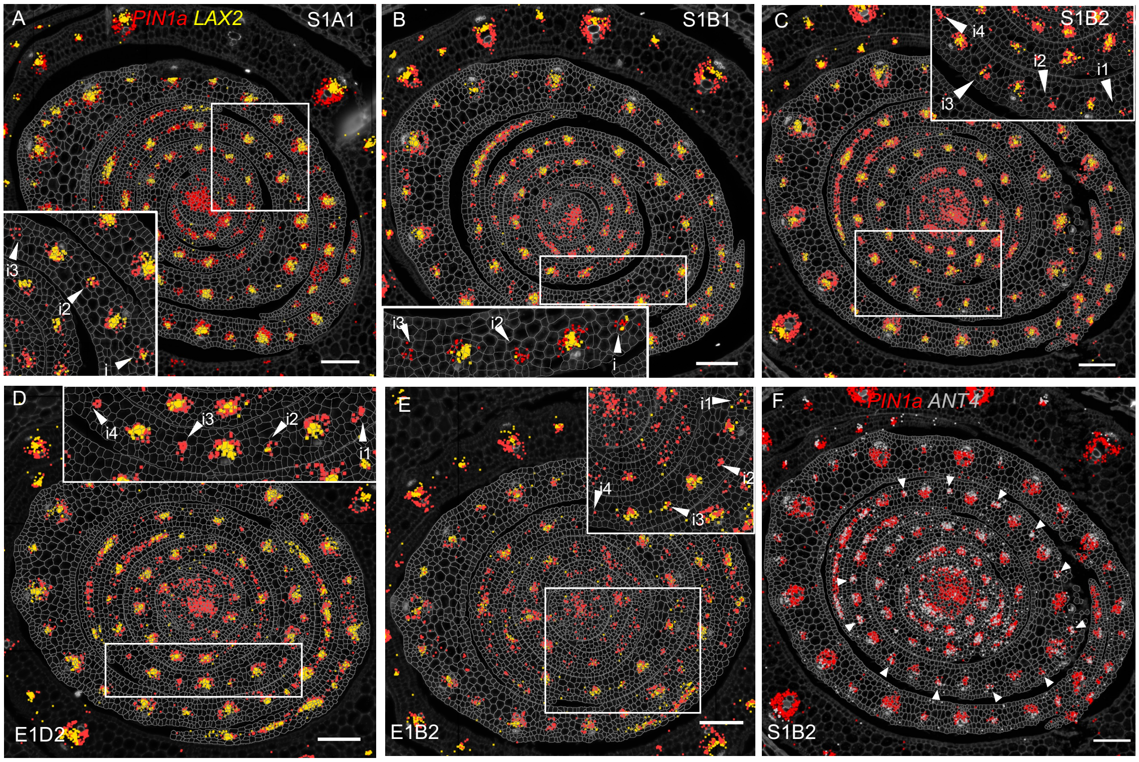

Fig. S7. *PIN1a* expression precedes that of *LAX2* across the medio-lateral axis. **A-E)** Transcript accumulation for *PIN1a* and *LAX2* is detected in both the sheath and blade and in both major and minor veins. Intermediate veins closer to the midrib accumulate *LAX2* before those closer to the leaf margin. In insets, intermediate veins are labelled with progressive numbers indicating the degree of proximity to the midrib. **F)** Unlike *LAX2*, *ANT4* is detected in all intermediate veins across the medio-lateral axis (white arrowheads). Scale bars = 100 µm.

Table S1. List of genes used for Molecular Cartography. Maize candidate regulators of vein development and Kranz anatomy were selected based on known function and/or mutant phenotype in monocot and dicot species. Orthology with known genes from eudicot or monocot systems is provided, alongside references to the origin of the gene names.

Gene_name	Gene_name Maize GDB	Gene ID (v3)	Gene ID (v4)	Gene ID (v5)	Origin of gene name (*supported by phylogeny)	Orthology	Variations
TML1	dot5	GRMZMG2150011	Zm00001d020037_T001	Zm00001eb309530	Vlad et al 2024*	AtWIP6	Previously refrred to as Zmdot1 or Zmdot5
TML2	–	GRMZM2G134998	Zm00001d005566_T001	Zm00001eb097850	Vlad et al 2024*	AtWIP6	Previously refrred to as Zmdot1h
SCR1	scro1	GRMZM2G131516	Zm00001d052380_T001	Zm00001eb195650	Hughes et al. 2019*	AtSCR	
SCR1h	Gras 37	GRMZM2G015080	Zm00001d005029_T001	Zm00001eb093670	Hughes et al. 2019*	AtSCR	
SHR1	Gras 58	GRMZM2G132794	Zm00001d029607_T001	Zm00001eb020840	Schuler et al. 2017*	AtSHR; OsSHR2	
SHR2	Gras 19	GRMZM2G172657	Zm00001d021973_T001	Zm00001eb326020	Schuler et al. 2017*	AtSHR; OsSHR1	
SHR2h	Gras 85	GRMZM2G019060	Zm00001d006721_T001	Zm00001eb108090	Schuler et al. 2017*	AtSHR; OsSHR1	
RVN1	IDD10	GRMZM2G143723	Zm00001d039467_T001	Zm00001eb121010	Sedelnikova et al. 2018	–	
RVN1h	IDD 18	GRMZM2G046290	Zm00001d008842_T001	Zm00001eb337700	Sedelnikova et al. 2018	–	
ANT4	EREB161	GRMZM2G021573	Zm00001d048004_T002	Zm00001eb399440	Liu et al. 2019*	ANT1-4 all co-orthologs of AtANT1	
PIN1a	pin1	GRMZM2G098643	Zm00001d044812_T001	Zm00001eb372180	Matthes et al. 2019*	Co-ortholog AtPIN1, ortholog OsPIN1a	
PIN1d	pin4	GRMZM2G171702	Zm00001d052442_T001	Zm00001eb196240	O'Connor et al 2014*	Ortholog of OsPIN1c & OsPIN1d	Also named ZmPIN1d in many papers
LAX1	aic3	GRMZM2G149481	Zm00001d028401_T005	Zm00001eb010460	Matthes et al. 2019*	Co-ortholog AtLAX2, OsLAX2 & OsLAX4	Named ZmLAX2 in Liu et al 2022
LAX2	aic1	GRMZM2G129413	Zm00001d030310_T001	Zm00001eb026490	Matthes et al. 2019*	Co-ortholog AtLAX2, ortholog of OsLAX4	
LAX3	atl1	GRMZM2G127949	Zm00001d042809_T003	Zm00001eb147480	Matthes et al. 2019*	Co-ortholog AtAUX1 & AtLAX1, ortholog of OsLAX1	
LAX5	aic4	GRMZM2G067022	Zm00001d038275_T005	Zm00001eb288800	Matthes et al. 2019*	Co-ortholog AtAUX1 & AtLAX1, ortholog of OsLAX3	
HB52	hb52	–(AC187157.4_FG005)	Zm00001d008869_T001	Zm00001eb337970	TAIR	Ortholog of AtHB8 & AtHB15	
ARF4	arftf4	GRMZM2G034840	Zm00001d001945_T010	Zm00001eb067270	Matthes et al. 2019*	Co-ortholog of AtMP, OsARF11	
ARF29	arftf29	GRMZM2G086949	Zm00001d026540_T016	Zm00001eb433020	Matthes et al. 2019*	Co-ortholog of AtMP, OsARF11	
ARF16	arftf16	GRMZM2G028980	Zm00001d053819_T008	Zm00001eb207450	Matthes et al. 2019*	OsARF6	
ARF34	arftf34	GRMZM2G081158	Zm00001d031064_T006	Zm00001eb031700	Matthes et al. 2019*	Co-ortholog of AtARF6, ortholog of OsARF17	
ARF23	arftf23	GRMZM2G441325	Zm00001d038698_T007	Zm00001eb292830	Matthes et al. 2019*	Co-ortholog of AtARF3 & AtARF4, ortholog of OsARF14	
KN1	kn1	GRMZM2G017087	Zm00001d033859_T002	Zm00001eb055920	Hake et al. 1989	Class I KNOX	
MYB67	myb67	GRMZM2G089244	Zm00001d053060_T001	Zm00001eb201150	Maize GDB	–	
CYC4	cyc4	GRMZM2G310115	Zm00001d012560_T002	Zm00001eb369340	Maize GDB	Ortholog of AtCYCB1 genes	cyc7 in raw data
CYC7	cyc7	GRMZM2G073003	Zm00001d043164_T001	Zm00001eb150450	Maize GDB	Ortholog of AtCYCB1 genes	previously cyc4b, cyc4 in raw data
CYC3	cyc3	GRMZM2G073671	Zm00001d036360_T002	Zm00001eb272010	Maize GDB	Ortholog AtCYCB2 genes	

Table S2. Gene expression in P0 cells identified from the 'peripheral zone' cluster. Transcript levels and identification labels of cells co-expressing *LAX5* and *PIN1d* within the "peripheral zone" cluster (arrowheads, Fig. 2E-F). Cells in bold are localized within the P0 primordium (Fig. 2G).

Gene.ID	E1B2_M_P1_23	E1B2_M_P1_121	E1B2_M_P1_191	S1A1_M_P1_39	S1A1_M_P1_49	S1A1_M_P1_82	S1A1_M_P1_83	**S1A1_M_P1_88**	**S1A1_M_P1_95**	**S1A1_M_P1_100**	**S1A1_M_P1_124**	**S1A1_M_P1_127**	**S1A1_M_P1_150**	S1A1_M_P1_160	S1A2_M_P1_16	S1A2_M_P1_72	S1A2_M_P1_81	S1A2_M_P1_234	S1B1_M_P1_183
SHR2	0	0	0	0	0	0	0	0	0	0	0	0	0	0	0	0	0	0	0
TML2	0	0	0	0	0	0	0	0	0	0	0	0	0	0	0	0	0	0	0
PIN1a	0	0	1	0	0	3	0	1	3	6	0	0	3	0	1	0	0	0	0
SCR1h	0	0	0	0	1	1	0	0	0	0	0	0	0	0	0	0	0	0	1
RVN1	0	0	0	0	0	0	0	0	0	0	0	0	0	0	0	0	0	0	0
KN1	1	3	0	0	0	14	0	0	0	0	0	0	0	0	0	0	0	0	0
CYC3	1	0	0	0	0	0	0	0	1	2	0	1	0	0	0	0	0	0	0
CYC4	0	0	0	0	0	0	0	0	0	5	2	0	0	0	0	0	0	0	0
ANT4	0	0	0	2	0	0	3	0	0	5	1	0	4	6	0	0	2	0	0
PIN1d	0	0	0	0	2	0	7	6	5	16	2	3	10	4	0	2	1	3	1
LAX1	0	0	0	0	0	0	0	1	0	1	0	2	0	0	0	0	1	0	1
LAX3	0	0	1	1	0	8	2	7	1	9	2	3	8	0	0	1	2	2	0
LAX5	1	1	1	1	0	2	1	2	2	5	6	3	5	2	1	1	1	1	1
HB52	0	1	0	0	2	1	0	3	1	5	4	4	2	7	11	4	5	6	0
ARF4	1	1	3	0	0	7	4	1	2	0	5	4	0	0	3	1	1	6	0
ARF29	1	0	0	0	0	0	0	0	0	0	0	0	0	0	0	0	0	0	0
ARF16	0	0	0	0	3	8	2	5	4	2	5	6	1	6	0	0	0	5	1
ARF34	5	4	3	2	7	0	1	7	5	9	4	11	7	5	2	6	3	1	5
ARF23	0	0	0	0	6	1	3	3	10	21	12	14	13	3	1	0	4	2	1
MYB67	0	0	0	0	0	0	0	1	0	0	0	1	1	0	0	0	0	0	0
CYC7	1	0	1	1	7	0	0	3	15	21	27	14	2	3	9	0	1	0	0

Table S3. Lateral and intermediate vein width quantification across the medio-lateral axis of leaves three and four. Quantification was carried out on half-leaves of expanded leaves three and four of plants with identical ID numbers (n=5). Lateral veins are labelled with progressive numbers from the midrib to the leaf margin, where “L1” indicates the lateral vein closest to, but outside of, the midrib region. Intermediate vein number and width are quantified between pairs of lateral veins, with pair L1-L2 being the closest to the midrib. Whereas a general decrease in vein width is observed across the medio-lateral axis for both lateral and intermediate veins, lateral veins are consistently wider than intermediate veins across the whole axis.

LEAF 3				Lateral veins width (μm) - half leaf				# Intermediates - half leaf				Avg. intermediate veins width (μm) - half leaf			
Plant ID	Blade width (mm)	Blade length (mm)	# Laterals - half leaf	L1	L2	L3	L4	L1-L2	L2-L3	L3-L4	L4-margin	L1-L2	L2-L3	L3-L4	L4-margin
1	14.49	330	4	112.89	106	88.24	89.64	15	14	8	5	48.9	45	45.3	44.4
2	16.56	360	4	118.44	121.82	97.7	83.81	16	16	8	4	49.75	49.36	42.67	41.21
3	16.8	365	4	150.73	116.4	106.87	76.53	12	14	13	11	52.42	48.28	46.69	49.73
4	17.99	300	4	138.18	131.45	125.36	83.93	13	14	12	6	62.4	53.77	43.29	43.53
5	14.79	310	4	110.92	105.63	95.02	83.19	14	15	10	4	53.06	42.79	35.22	44.29
**Avg.**	**16.13**	**333.00**	**4.00**	**126.23**	**116.26**	**102.64**	**83.42**	**14.00**	**14.60**	**10.20**	**6.00**	**53.31**	**47.84**	**42.63**	**44.63**
stdev	1.46	29.07	0.00	17.44	10.95	14.35	4.65	1.58	0.89	2.28	2.92	5.38	4.22	4.44	3.13

LEAF 4				Lateral veins width (μm) - half leaf					# Intermediates - half leaf					Avg. intermediate veins width (μm) - half leaf				
Plant ID	Blade width (mm)	Blade length (mm)	# Laterals - half leaf	L1	L2	L3	L4	L5	L1-L2	L2-L3	L3-L4	L4-L5	L5-margin	L1-L2	L2-L3	L3-L4	L4-L5	L5-margin
1	20.33	460	5	112.3	140.05	124.7	107.75	87.82	14	16	14	8	9	49.4	43.89	48.77	57.43	51.09
2	24.1	460	5	147.23	153.06	137.04	120.53	82.18	14	15	13	8	4	53.11	49.98	51.27	48.67	56.02
3	23.19	470	5	137.34	122.93	125.94	103.3	83.93	15	15	14	11	9	47.86	51.97	52.78	48.59	44.51
4	20.89	440	5	118.7	128.56	153.13	115.5	89.7	15	17	17	8	5	48.58	57.1	54.66	45.65	55.88
5	22.33	420	5	120.1	127.34	130.81	98.77	101.37	10	12	14	10	7	65.58	56.91	55.98	58.68	45.7
**Avg.**	**22.17**	**450.00**	**5.00**	**127.13**	**134.39**	**134.32**	**109.17**	**89.00**	**13.60**	**15.00**	**14.40**	**9.00**	**6.80**	**52.91**	**51.97**	**52.69**	**51.80**	**50.64**
stdev	1.57	20.00	0.00	14.57	12.20	11.58	8.86	7.54	2.07	1.87	1.52	1.41	2.28	7.37	5.48	2.83	5.85	5.44

Dataset S1 (separate file). Unprocessed and processed (normalised and scaled) transcript counts for stages M-P1, P2, P3, P4. Cells are labelled based on the section of origin (E1B2, E1D2, S1A1, S1A2, S1B1 or S1B2), and the plastochron stage under analysis (M-P1, P2, P3, P4). These data were used to generate Figs. 2, 4 and 5.

Dataset S2 (separate file). Molecular Cartography data clustering. Identities are labelled as outlined in Figs. 2A, 4A, 5A-B and S5. Cell labels as in SI Appendix, Dataset S1.

Dataset S3 (separate file). Unprocessed transcript counts for the analysis shown in SI Appendix, Fig. S5.

Dataset S4 (separate file). Normalised and scaled data obtained from Dataset S3. Refers to the analysis shown in SI Appendix, Fig. S5.

SI References

1. P. Glenn, *et al.*, Wheat bZIPC1 interacts with FT2 and contributes to the regulation of spikelet number per spike. *Theoretical and Applied Genetics* **136**, 1–20 (2023).
2. D. Vlad, *et al.*, The WIP6 transcription factor TOO MANY LATERALS specifies vein type in C_4 and C_3 grass leaves. *Current Biology* **34**, 1–17 (2024).
3. Y. Hao, *et al.*, Integrated analysis of multimodal single-cell data. *Cell* **184**, 3573-3587.e29 (2021).
4. Wei Tayun, Simko Viliam, R package “corrplot”: visualization of a correlation matrix. (Version 0.92) (2021).
5. M. L. Schuler, O. V. Sedelnikova, B. J. Walker, P. Westhoff, J. A. Langdale, SHORTROOT-Mediated increase in stomatal density has no impact on photosynthetic efficiency. *Plant Physiol* **176**, 757–772 (2018).
6. D. Jackson, B. Veit, S. Hake, Expression of maize KNOTTED1 related homeobox genes in the shoot apical meristem predicts patterns of morphogenesis in the vegetative shoot. *Development* **120**, 405–413 (1994).
